# Investigating the Efficacy and Safety of Thalidomide for Treating Patients With *ß*-Thalassemia: A Meta-Analysis

**DOI:** 10.3389/fphar.2021.814302

**Published:** 2022-01-11

**Authors:** Yanfei Lu, Zhenbin Wei, Gaohui Yang, Yongrong Lai, Rongrong Liu

**Affiliations:** Department of Hematology, The First Affiliated Hospital of Guangxi Medical University, Nanning, China

**Keywords:** thalidomide, *ß*-thalassemia, meta-analysis, hemoglobin level, therapy

## Abstract

At present, the main therapies for *ß*-thalassemia patients include regular blood transfusion and iron chelation, associating with a number of limitations. Thalidomide, a fetal hemoglobin (HbF) inducer that promotes γ-globin gene expression, has been reported to be effective for *ß*-thalassemia. Thus, this meta-analysis was conducted to assess the efficacy and safety of thalidomide for treating patients with *ß*-thalassemia. We searched the related studies from eight databases published from inception until December 1, 2021. The R 4.0.5 language programming was used to perform meta-analysis. After screening of retrieved articles, 12 articles were included that enrolled a total of 451 patients. The Cochrane Collaboration risk assessment tool was used to evaluate the quality and the bias risk of the randomized controlled trials (RCTs), and non randomized trials were assessed using Newcastle-Ottawa Scale (NOS). After treatment with thalidomide, the pooled overall response rate (ORR) was 85% (95% confidence interval (CI): 80–90%), and the pooled complete response rate (CRR) was 54% (95% confidence interval: 31–76%). Compared with the placebo group, the thalidomide group had higher odds of overall response rate (odds ratio = 20.4; 95% CI: 6.75–61.64) and complete response rate (odds ratio = 20.4; 95% CI: 6.75–61.64). A statistically significant increase in hemoglobin level and HbF level after treatment, while there was no statistically significant difference in adult hemoglobin (HbA) level, spleen size, and serum ferritin. According to the results of ORR and CRR, transfusion-dependent thalassemia (TDT) patients showed remarkable efficacy of thalidomide, 83 and 52% respectively. So we analyzed 30 transfusion-dependent thalassemia patients from three studies and found that the most frequent *ß*-globin gene mutations were CD41-42 (-TCTT), while response to thalidomide did not show any statistically significant relationship with XmnI polymorphism or CD41-42 (-TCTT) mutation. About 30% of patients experienced mild adverse effects of thalidomide. Collectively, thalidomide is a relatively safe and effective therapy to reduce the blood transfusion requirements and to increase Hb level in patients with *ß*-thalassemia.

## Introduction

β-thalassemia comprises a group of hereditary hematological disorders characterized by abnormalities in the synthesis of the *ß* chains of hemoglobin. With the migration of populations, nearly 5% of the world’s population carry mutations of globin genes (Angastiniotis and Modell, 1998). It was estimated that about 1.5% of the global population (80–90 million people) are carriers of *ß*-thalassemia (Modell and Darlison, 2008). The basic defect in *ß*-thalassemia is an imbalanced relationship between the alpha globin and beta globin chains caused by a reduced or absent production of *ß*-globin chains. An excess unmatched *a*-globin chain precipitates in erythropoiesis, resulting in elevated mechanical stresses and oxidative damage to erythropoiesis in patients with *ß*-thalassemia. The clinical severity of *ß*-thalassemia is related to the extent of an imbalanced relationship between the alpha globin and non-alpha globin chains, resulting in variable phenotypes, ranging from severe anemia to clinically asymptomatic individuals.

At present, *ß*-thalassemia seriously threatens public health, and the main therapies for patients with *ß*-thalassemia include regular blood transfusion and iron chelation. However, long-term blood transfusion can cause iron overload and also spread transfusion-related infectious diseases. Adverse effects (AEs) of iron-chelating agents have also been reported. Because of the decrease in blood donation rates caused by the coronavirus disease 2019 pandemic, the global demand of blood products exceeds supply. Blood transfusion in patients with *ß*-thalassemia is associated with difficulties. Hematopoietic stem cell transplantation (HSCT) is the primary method to cure patients with *ß*-thalassemia, while due to the high level of HLA polymorphism, the major obstacle in the HSCT is to find a donor with perfectly matched HLA antigens. Recently, gene therapy and gene editing techniques have assisted clinicians to treat *ß*-thalassemia, while they have not been popularized globally. In addition to traditional treatments, novel therapies for *ß*-thalassemia have been presented. Activin receptor ligand traps are also effective in treating anemia associated with *ß*-thalassemia (Suragani et al., 2014). In 2019, the United States Food and Drug Administration approved luspatercept for treating anemia in adult patients with *ß*-thalassemia. Other therapies, such as Janus kinase 2 (JAK2) inhibitor, have been previously reported to improve iron dysregulation and reduce synthesis of *a*-globin chain.

Despite advances achieved in the therapy of *ß*-thalassemia patients, maintaining a high hemoglobin (Hb) level is still a main challenge. Therefore, there is an urgent need for novel, effective, and safe treatments for *ß*-thalassemia. Fetal hemoglobin (HbF) inducers have markedly attracted clinicians’ attention in recent years, and thalidomide was reported as a promising drug in the treatment of *ß*-thalassemia. Thalidomide was originally used to stop vomiting in pregnancy, while it has been withdrawn from the market because of its teratogenic effects. It has been reused as an immunomodulatory and anti-angiogenic drug to treat autoimmune diseases. To date, few retrospective studies have pointed out that thalidomide, as a strong HbF inducer, could improve the clinical symptoms and the quality of life of patients with *ß*-thalassemia.

The efficacy of thalidomide in human models has not been comprehensively determined, and there is a certain degree of uncertainty. The present meta-analysis aimed to assess the efficacy and safety of thalidomide in treating patients with *ß*-thalassemia.

## Methods

### Search Strategy

We searched the related studies from PubMed, Web of Science, embase, Cochrane Library, China Biology Medicine disc, China National Knowledge Infrastructure, Wanfang, and VIP Data Knowledge Service Platform published from inception until December 1, 2021. The following terms were searched: (“Thalidomide”) OR (Sedoval [All Fields]) OR (Thalomid)) AND ((“Thalassemia”) OR (Thalassemia [All Fields])). The protocol has been registered in the International Prospective Register of Systematic Reviews (PROSPERO; Registration No. CRD42014010138).

### Inclusion and Exclusion Criteria

The inclusion criteria were as follows: 1) studies conducted prospectively or retrospectively; 2) patients who were diagnosed with *ß*-thalassemia; and 3) if a study included two or more groups to compare thalidomide with other treatments for primary diseases, such as hydroxyurea, erythropoietin, etc., the group of treatment with thalidomide was included.

The exclusion criteria were as follows: 1) thalidomide combined with other treatments for primary diseases; 2) patients who underwent therapies for less than 3 months. If a study included patients who underwent therapies for ≥3 and <3 months, patients who underwent therapies for <3 months were excluded; 3) case reports, reviews, conference abstracts, or *in vitro* studies; or 4) studies with incomplete data.

### Assessment of the Quality of Included Studies

The Cochrane Collaboration risk assessment tool was used to evaluate the quality and the bias risk of the randomized controlled trials (RCTs). It includes selection bias (random sequence generation and allocation concealment); blinding of participants, personnel, and outcomes assessment; completeness of data; selective outcome reporting; and other potential biases. Based on above domains, we categorised studies as having “low”, “unclear” or “high” bias risk. The Newcastle-Ottawa Scale (NOS) was used to assess the non randomized studies. The NOS evaluates three quality parameters (selection, comparability, and outcome) divided across eight specific items. Each item on the scale is scored from one point, except for comparability, which can be adapted to the specific topic of interest to score up to two points. Thus, the maximum for each study is 9. Studies with <3 points were at a high risk of bias and were excluded.

### Selection of Eligible Studies and Data Extraction

Two researchers independently searched and selected the eligible studies. Titles and abstracts of retrieved articles were reviewed to exclude duplicate and irrelevant studies. Then, the full-texts of the remaining articles were read, and eligible studies that met the inclusion criteria but did not meet the exclusion criteria were included. Then, the following data were extracted: the first author’s full-name, year of publication, research site (a place where scholars conducted research), type of study, type of disease, utilization of blood transfusion, follow-up data, age of patients, sample size, type of intervention. Any disagreements were resolved by discussion with a third researcher.

### Outcomes and Statistical Analysis

The primary outcomes were overall response rate (ORR), complete response rate (CRR), and Hb level. The secondary outcomes were HbF level, adult hemoglobin (HbA) level, spleen length, and adverse effects (AEs) of thalidomide. ORR was defined as ≥ 50% reduction in transfusion requirement or an increase in Hb level ≥1.0 g/dl for transfusion-dependent thalassemia (TDT) patients after thalidomide treatment. For non-transfusion-dependent thalassemia (NTDT) patients, ORR was defined as an increase in Hb level ≥1.0 g/dl. CRR was defined as complete cessation of regular transfusion.

The R 4.0.5 language programming was used to perform meta-analysis. We used the I^2^ statistic to test the heterogeneity among the included studies. I^2^ ≥ 50% represented a substantial heterogeneity, thus, the random-effects model was used to perform the analysis; otherwise, the fixed-effects model was utilized. The sensitivity analysis was conducted to explore whether the results are sensitive to exclusion of low-quality studies. Egger’s regression test was used to explore the presence of publication bias.

The continuous variables (Hb, HbF, HbA, spleen size, and serum ferritin (SF)) were expressed as mean ± standard deviation (SD). Data presented with 95% confidence intervals (95% CIs) were analyzed to estimate the efficacy and AEs. We obtained individual participant data from the principal investigator of Yang et al.‘s study. Statistical analyses were performed using SPSS 26.0. The Chi-square test or Fisher’s exact test was used to analyze the association of response to thalidomide with XmnI polymorphism and genotypes. *p-value* < 0.05 was considered statistically significant.

## Results

### Study Characteristics

**FIGURE 1:**
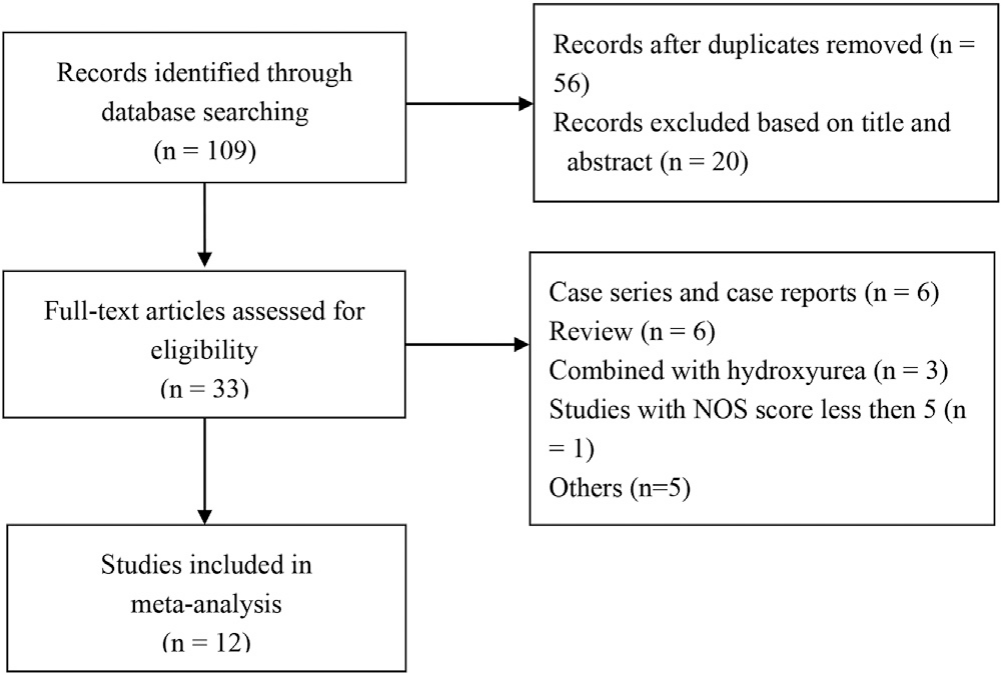
Flowchart of study selection process. NOS, Newcastle-Ottawa Scale.

A total of 109 articles were retrieved from the search strategy. After screening of title, abstract, and full-text of those articles, 12 articles were included for analysis (Chen et al., 2017; Li et al., 2018; Ren et al., 2018; Jain et al., 2019; Begum et al., 2020; Islam et al., 2020; Nag et al., 2020; Sen et al., 2020; Yang et al., 2020; Yassin, 2020; Chen et al., 2021; Li et al., 2021), which enrolled 451 patients. Jain et al. conducted a RCT and compared hydroxyurea arm with thalidomide arm, while only thalidomide arm was included in the present meta-analysis (Jain et al., 2019). One RCT study and 11 single-arm studies (4 retrospective and eight prospective studies) were included, of which 6 studies were conducted in China, three in India, two in Bangladesh, and one in Iraq. Figure 1 shows flowchart of study selection process according to PRISMA guidelines. Characteristics of the included studies are summarized in Table 1.

**TABLE 1:** Characteristics of the included studies.

Authors	Year	Country	Design	Disease	Population	F/U, month (range)	Age, year (range)	N (M/F)	Intervention
Chen	2017	China	Retrospective study	β-TM	NTDT and TDT	12	24 (13–74)	9 (5/4)	Thalidomide (the dose started at 50 mg/d and increased to 200 mg/d as tolerated)
Li	2018	China	Prospective study	β-TM	NTDT and TDT	TDT: > 6 NTDT: > 3	29.4 (25–35)	7 (4/3)	Thalidomide (the dose was 50 mg/d)
Ren	2018	China	Prospective study	β-TM	NTDT	3	25.6 (18–36)	14 (8/6)	Thalidomide (the dose was 50 mg/d)
Jain	2019	India	Prospective study	HbE-β-TM	NTDT	6	27 (7–45)	15 (8/7)	Thalidomide (the dose was 50 mg/d)
Yassin	2020	Iraq	Prospective study	β-TM	NTDT and TDT	8–36 (15)	9 (3–43)	37 (21/16)	Thalidomide (the dose was 2–10 mg/kg/d, and a initial dose of 3 mg/kg/d adjusted to nearest 50 mg/d was used)
Nag	2020	India	Retrospective study	HbE-β-TM	TDT	3	20	21 (7/14)	Thalidomide (age ≤12Y: the dose was 50 mg/d; age >12Y: the dose was 100 mg/d)
Islam	2020	Bangladesh	Retrospective study	HbE-β-TM	TDT	16 (3–38)	15 (3–49)	50 (26/24)	Thalidomide (the dose was 50–100 mg/d)
Begum	2020	Bangladesh	Prospective study	HbE-β-TM	TDT	32	10 (3–24)	51 (28/23)	Thalidomide (the dose was 2–5 mg/kg/d)
Sen	2020	India	Prospective study	HbE-β-TM and β-TM	NTDT and TDT	3–11 (7.1 ± 3.3)	15 (2–44)	9 (6/3)	Thalidomide (the dose was 50 mg/d)
Yang	2020	China	Prospective study	β-TM	NTDT and TDT	3–37	27.2 (15–45)	62 (27/35)	Thalidomide (the initial dose was 50 mg/d, and dose of 100 mg/d was given to patients needing blood transfusions at least twice a month)
Li	2021	China	Retrospective study	β-TM	TDT	(14.6 ± 9.6)≥6	10 (5–18)	77 (45/32)	Thalidomide (the dose was 2.5–4 mg/kg/d)
Chen	2021	China	Prospective study	β-TM	TDT	≥3	18.4	99 (62/37)	Thalidomide (initial dose of 100 mg/d and escalated to 150 mg/d in 3 days if no adverse effects were reported)

F/U, follow-up; M/F, male/female; N, sample size; Y, year; TM, thalassemia; NTDT, non-transfusion-dependent thalassemia, TDT, transfusion-dependent thalassemia.

## Quality Assessment

The included RCT study was of high-quality through the Cochrane Collaboration risk assessment tool (Supplementary Figures S1,2). Using the NOS, we excluded a study with low quality. All the single-arm studies ranged from five to eight points, which indicated that their quality ranged from medium to high (Supplementary Table S1).

## Outcomes

### ORR

**FIGURE 2:**
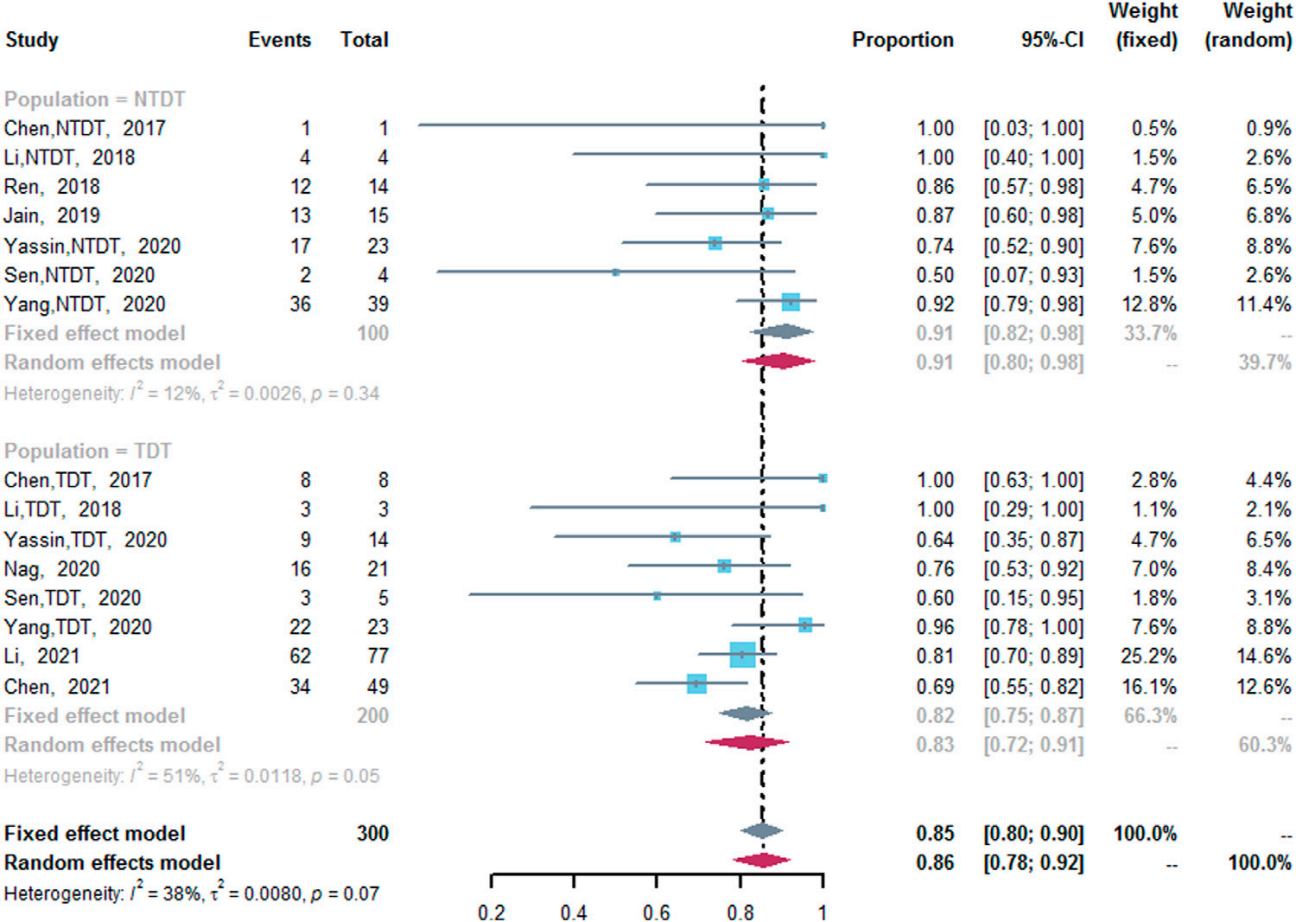
A forest plot illustrating overall response rate in population-based subgroups.

ORR was assessed in 10 studies (Chen et al., 2017; Li et al., 2018; Ren et al., 2018; Jain et al., 2019; Nag et al., 2020; Sen et al., 2020; Yang et al., 2020; Yassin, 2020; Chen et al., 2021; Li et al., 2021). Five studies included both TDT and NTDT patients (Chen et al., 2017; Li et al., 2018; Sen et al., 2020; Yang et al., 2020; Yassin, 2020), and the outcomes of TDT and NTDT patients were separated in the original manuscripts, so we analyzed the data in these studies respectively. Chen et al. conducted a RCT included thalidomide group and placebo group (Chen et al., 2021). We not only included the thalidomide group alone to assess the pooled ORR and CRR, but also compared the ORR and CRR of the thalidomide group and the placebo group. The pooled ORR was 85% (95% CI: 80–90%, I^2^ = 38%, *p* = 0.07), and the pooled ORR values in two subgroups were different. Seven studies reported the ORR of NTDT patients after the treatment of thalidomide (Chen et al., 2017; Li et al., 2018; Ren et al., 2018; Jain et al., 2019; Sen et al., 2020; Yang et al., 2020; Yassin, 2020), in which the ORR was 91% (95% CI: 82–98%, I^2^ = 12%, *p* = 0.34). In addition, eight studies reported the ORR of TDT patients after the treatment of thalidomide (ORR, 83%; 95% CI: 72–91%, I^2^ = 51%, *p* = 0.05) (Figure 2) (Chen et al., 2017; Li et al., 2018; Nag et al., 2020; Sen et al., 2020; Yang et al., 2020; Yassin, 2020; Chen et al., 2021; Li et al., 2021). Compared with the placebo group, the thalidomide group had higher odds of ORR (odds ratio = 20.4; 95% CI: 6.75–61.64) (Supplementary Figure S3) (Chen et al., 2021).

### CRR

**FIGURE 3:**
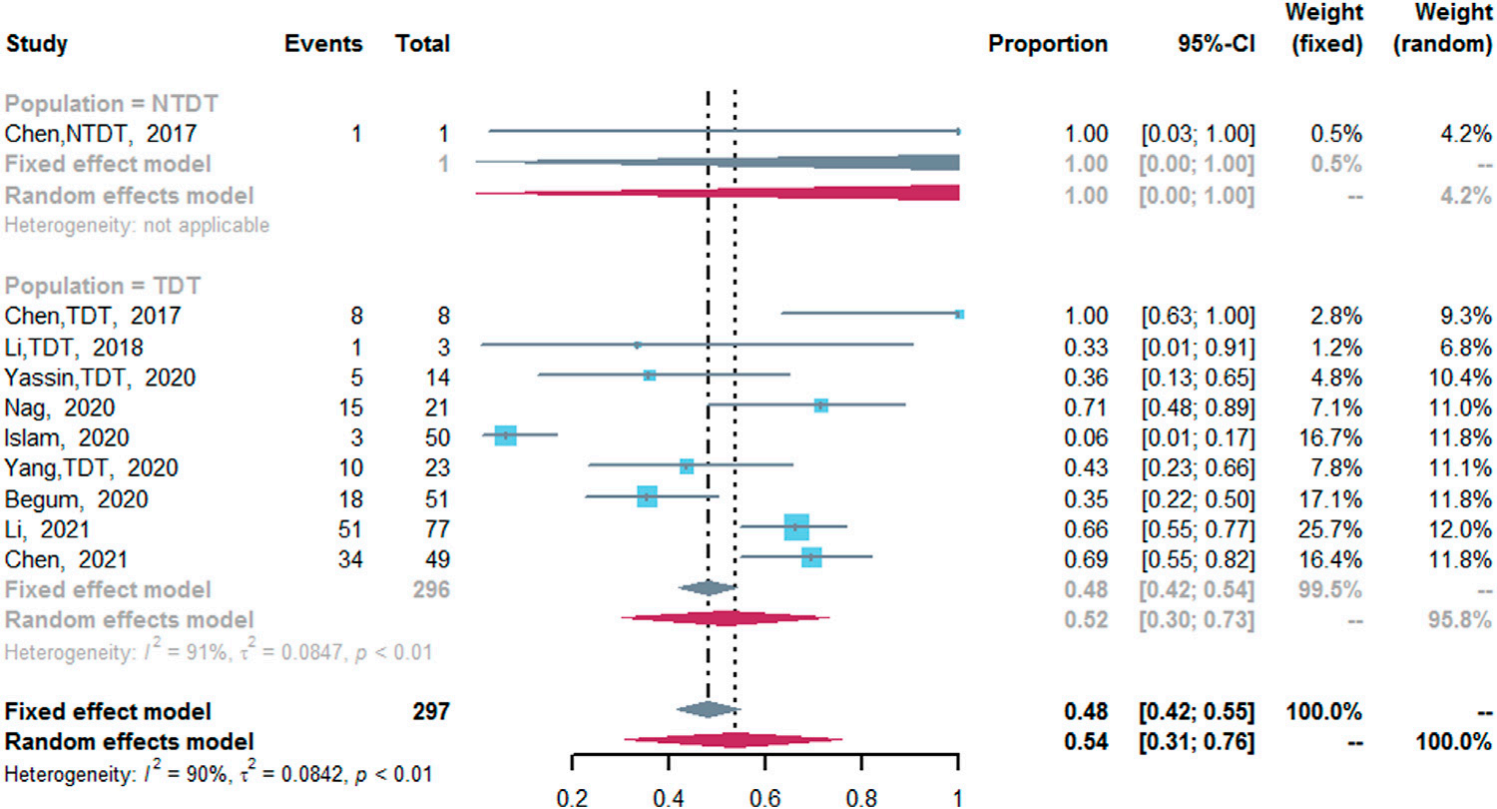
A forest plot illustrating complete response rate in population-based subgroups.

CRR was assessed in nine studies (Chen et al., 2017; Li et al., 2018; Begum et al., 2020; Islam et al., 2020; Nag et al., 2020; Yang et al., 2020; Yassin, 2020; Chen et al., 2021; Li et al., 2021). The pooled CRR was 54% (95% CI: 31–76%, I^2^ = 90%, *p* < 0.01). Nine studies reported the CRR of TDT patients after the treatment of thalidomide (Chen et al., 2017; Li et al., 2018; Begum et al., 2020; Islam et al., 2020; Nag et al., 2020; Yang et al., 2020; Yassin, 2020; Chen et al., 2021; Li et al., 2021), in which the CRR was 52% (95% CI: 30–73%, I^2^ = 91%, *p* < 0.01). However, only one study reported the CRR of NTDT patients after the treatment of thalidomide (CRR, 100%; 95% CI: 0–100%) (Figure 3) (Chen et al., 2017). In addition, compared with the placebo group, the thalidomide group had higher odds of CRR (odds ratio = 20.4; 95% CI: 6.75–61.64) (Supplementary Figure S4) (Chen et al., 2021).

### Hb Level (g/dl)

**FIGURE 4:**
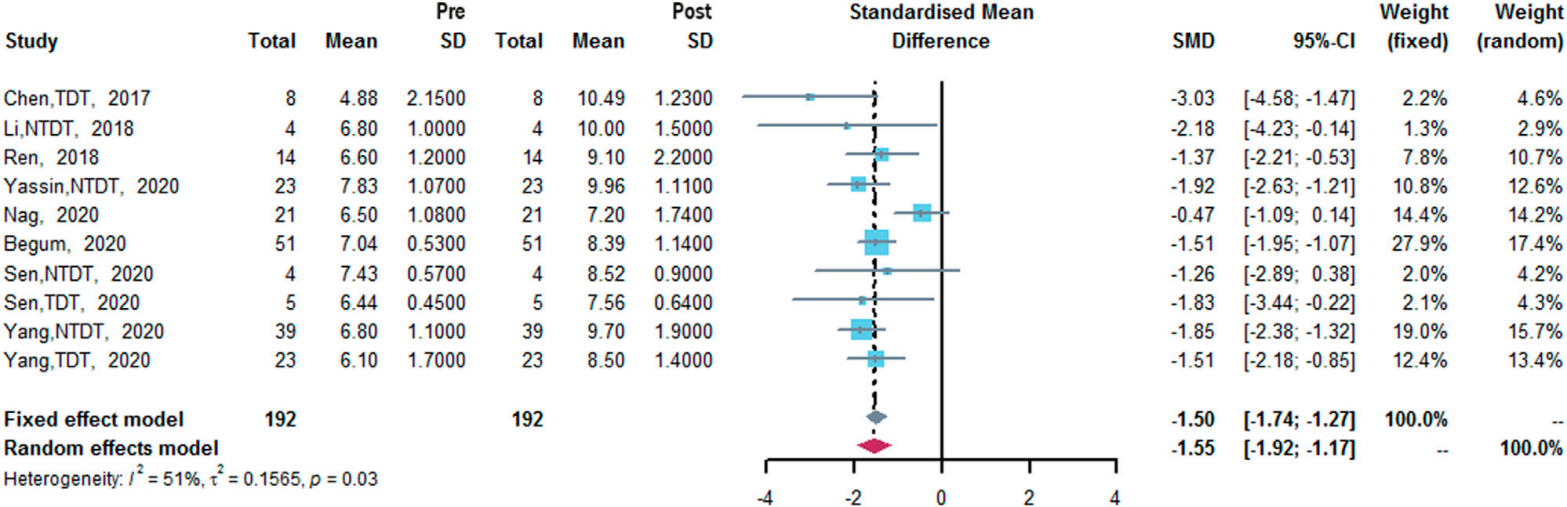
A forest plot illustrating Hb level (g/dl) after the treatment of thalidomide in population-based subgroups.

Eight studies measured Hb level (Chen et al., 2017; Li et al., 2018; Ren et al., 2018; Begum et al., 2020; Nag et al., 2020; Sen et al., 2020; Yang et al., 2020; Yassin, 2020). The results of meta-analysis showed that after treatment of thalidomide, a statistically significant increase of mean Hb level from baseline was 1.55 g/dl (95% CI: 1.17–1.92 g/dl). Heterogeneity was moderate (I^2^ = 51%, *p* = 0.03) (Figure 4).

### HbF Level (%)

**FIGURE 5:**
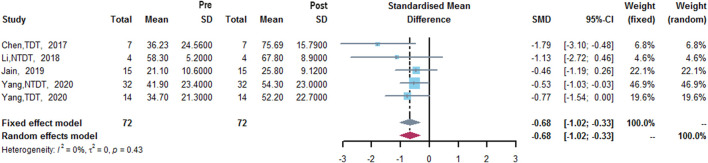
A forest plot illustrating HbF level (%) after the treatment of thalidomide.

Four studies measured HbF level (Chen et al., 2017; Li et al., 2018; Jain et al., 2019; Yang et al., 2020). The results of meta-analysis showed that after treatment with thalidomide, a statistically significant increase of mean HbF level from baseline was 0.68% (95% CI: 0.33–1.02%). Heterogeneity was low (I^2^ = 0%, *p* = 0.43) (Figure 5).

### HbA Level (g/dl)

Two studies measured HbA level (Li et al., 2018; Ren et al., 2018). The results of meta-analysis revealed that after the treatment of thalidomide, the change in HbA level from baseline was not statistically significant, 0.196 g/dl (95% CI: 0.463–0.855 g/dl). Heterogeneity was low (I^2^ = 0%, *p* = 0.42) (Supplementary Figure S5).

### Spleen Length (cm)

Two studies measured spleen length (Chen et al., 2017; Nag et al., 2020). The results of meta-analysis indicated that after the treatment of thalidomide, the change in spleen length from baseline was not statistically significant, 0.19 cm (95% CI: −0.85–1.23 cm). Heterogeneity was moderate (I^2^ = 68%, *p* = 0.08) (Supplementary Figure S6).

### SF Level (ng/ml)

Two studies measured SF concentration (Begum et al., 2020; Yang et al., 2020). The results of meta-analysis showed that after the treatment of thalidomide, the change in SF level from baseline was not statistically significant, 0.03 ng/ml (95% CI: −0.25–0.31 ng/ml). Heterogeneity was moderate (I^2^ = 48%, *p* = 0.15) (Supplementary Figure S7).

### AEs of Thalidomide

**FIGURE 6:**
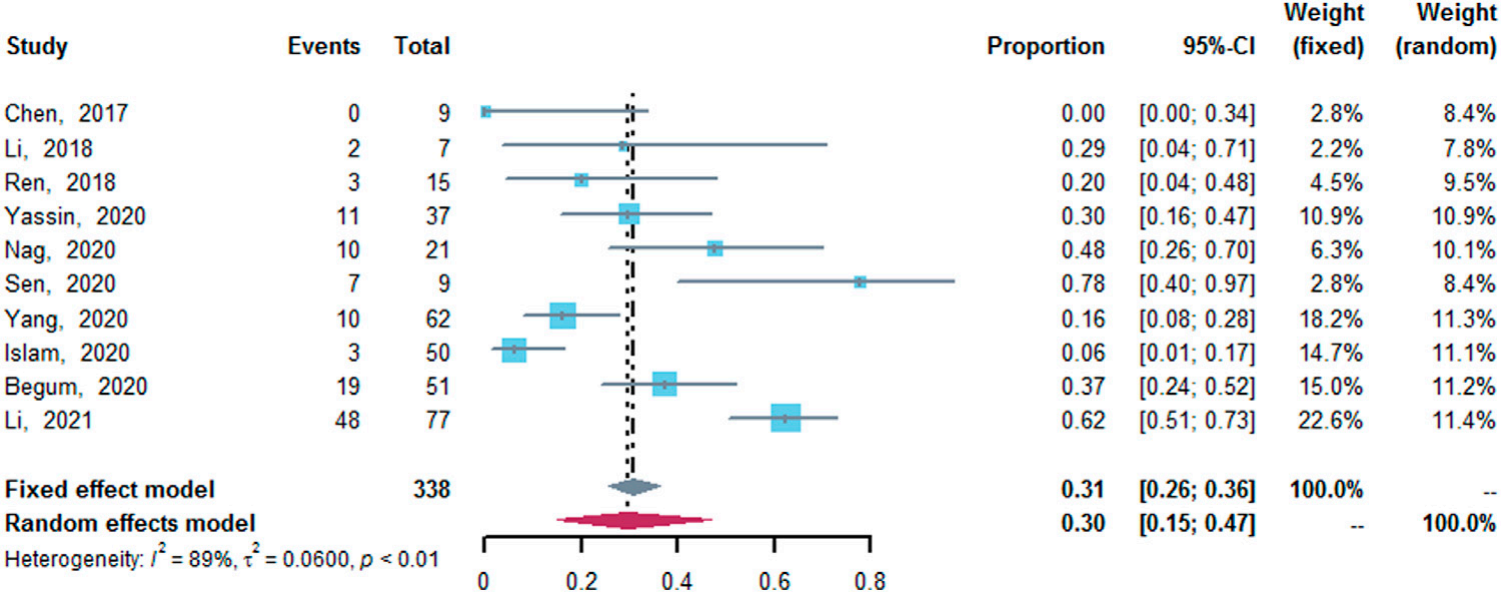
A forest plot illustrating adverse effects of thalidomide.

Ten studies (Chen et al., 2017; Li et al., 2018; Ren et al., 2018; Begum et al., 2020; Islam et al., 2020; Nag et al., 2020; Sen et al., 2020; Yang et al., 2020; Yassin, 2020; Li et al., 2021), which enrolled a total of 338 patients, reported AEs of thalidomide. The results of meta-analysis revealed that about 30% (95% CI: 15–47%, I^2^ = 89%, *p* < 0.01) of patients suffered from AEs of thalidomide. Among the included studies, except for three patients with peripheral neurotoxicity and/or headache (Yang et al., 2020; Li et al., 2021), one patient with central venous thrombosis and one patients with seizure (Li et al., 2021), the reported AEs were mild, and common AEs included constipation, somnolence, high alanine aminotransferase (ALT) level, and rash (Figure 6).

### Sensitivity Analysis and Publication Bias

To analyze heterogeneity, we conducted sensitivity analysis of pooled outcomes, and the reliability of pooled outcomes was confirmed, except for Hb level (Table 2). After removing Nag et al.‘s study (Nag et al., 2020), the results showed that the I^2^ was reduced, with a re-estimated mean change in Hb level from baseline was 1.68 g/dl (95% CI: 1.42–1.93 g/dl, I^2^ = 0%, *p* = 0.69) (Supplementary Figure S8). No significant publication bias was identified in Egger’s Test (Table 2).

**TABLE 2:** Results of sensitive analysis and tests of publication bias.

Outcome	No. of studies	Variations in sensitivity analyses	Effects model	Egger’s test for publication bias (*p value*)
ORR	10	NS	Fixed	0.818
CRR	9	NS	Random	—
Hb level	8	S	Random	—
HbF level	4	NS	Fixed	—
HbA level	2	NS	Fixed	—
Spleen length	2	NS	Random	—
SF level	2	NS	Fixed	—
AEs	10	NS	Random	0.818

no Egger’s test assessed because the number of included studies was <10; NS: no significant variations introduced; S: significant variations introduced.

## Discussion

In humans, *ß*-like-globin genes gradually express *e*-globin gene, *γ*-globin gene, and γ-globin gene at distinct stages of development through a process termed “hemoglobin switching”. γ-globin gene expresses γ-globin and forms HbF (*α*_2_γ_2_) with *a*-globin, and HbF is the most important hemoglobin in early fetal development. Around the time of birth, fetal *γ*-globin expression is extinguished and the adult γ-globin gene is activated (Stamatoyannopoulos, 2005). When carriers have mutant *ß*-globin gene, the synthesis of HbA (*α*_2_β_2_) decreases and *a* globin and *ß* globin chains are imbalanced, then the clinical symptoms associated with *ß*-thalassemia occur. The severity of disease (β-thalassemia) expression is mainly related to the degree of the unmatched *a*-globin chain. While scientists observed that *ß*-thalassemia patients with a high level of HbF had lower degree of anemia and were mainly diagnosed with NTDT (Winichagoon et al., 2000). Hereditary persistence of fetal hemoglobin (HPFH) is a condition that naturally occurs and is characterized by a considerable elevation of HbF level in adult red blood cells. Individuals with compound heterozygous *ß*-thalassemia and HPFH have milder clinical manifestations (Papadakis et al., 2002). This clinical benefit of increased HbF level leads to the emergence of numbers of HbF inducers. The earlier HbF inducers include hydroxyurea, DNA methyltransferase (DNMT) inhibitor, histone deacetylase inhibitor agents (Witt et al., 2003; Olivieri et al., 2011; Karimi et al., 2021). Hydroxyurea is the most widely used HbF inducer in patients with moderate and severe *ß*-thalassemia (Gambari and Fibach, 2007; Testa, 2009). A number of studies demonstrated that hydroxyurea appears to be effective, well tolerated, and associated with mild and transient adverse events for thalassemia patients (Haghpanah et al., 2018; Karimi et al., 2021). Some studies also reported that the efficacy of hydroxyurea declined in hematological response after treatment for 12 months (Mancuso et al., 2006; Rigano et al., 2010). DNMT inhibitors and histone deacetylase inhibitors were mainly tested in *vitro* or clinical trials and their safety and efficacy have not been widely demonstrated (Shearstone et al., 2016; Kalantri et al., 2018).

Now, thalidomide, as a novel HbF inducer, is an emerging treatment option for *ß*-thalassemia. A number of scholars pointed out that thalidomide induced γ-globin mRNA expression in a dose-dependent manner, while it had no effect on *ß*-globin expression (Aerbajinai et al., 2007). Several small case series and clinical trials (12 single-arm and 3 randomized controlled trials) have reported the efficacy and safety of thalidomide in patients with *ß*-thalassemia (Chen et al., 2017; Kalra et al., 2017; Li et al., 2018; Ren et al., 2018; Jain et al., 2019; Ricchi et al., 2019; Begum et al., 2020; Islam et al., 2020; Javed et al., 2020; Nag et al., 2020; Sen et al., 2020; Yang et al., 2020; Yassin, 2020; Chandra et al., 2021; Chen et al., 2021; Jain et al., 2021; Li et al., 2021). Chen et al. conducted the largest prospective study enrolled 99 patients has shown remarkable response to thalidomide treatment for patients with *ß*-thalassemia (Chen et al., 2021). Based on these studies, we conducted the present meta-analysis. According to the results, thalidomide increased Hb level by elevating HbF level, and the clinical symptoms of patients with *ß*-thalassemia were improved after treatment, which were consistent with previous studies (Fard et al., 2014). Furthermore, the efficacy of thalidomide for treating *ß*-thalassemia was markedly higher than that of hydroxyurea (Algiraigri et al., 2017a; Algiraigri et al., 2017b), and several included studies with the follow-up over 12 months demonstrated the stable efficacy of thalidomide (Islam et al., 2020; Yang et al., 2020; Yassin, 2020; Chen et al., 2021). About the effective dose of thalidomide, the included studies were given doses of thalidomide from 50 to 200 mg/day, while there was no obvious differences in increasing Hb level and improving clinical symptoms for *ß*-thalassemia between dose of 50 mg/d and 200 mg/d (Chen et al., 2017), and enormous doses of thalidomide can cause significant side effects, therefore, the low toxicity dose of 50 mg/d of thalidomide will meet the goal of improving anemia in patients with thalassemia clinically (Chen et al., 2017; Li et al., 2018; Ren et al., 2018; Jain et al., 2019; Islam et al., 2020; Nag et al., 2020; Sen et al., 2020; Yang et al., 2020). Interestingly, there was narrow difference of ORR between NTDT and TDT patients in this meta-analysis (91 versus 83%), and about 52% of TDT patients became completely transfusion free. It showed remarkable efficacy of thalidomide in TDT patients, and similar result was observed in the latest RCT study, which was encouraging. So we analyzed 30 TDT patients from three studies and found that the most frequent *ß*-globin gene mutations were CD41-42 (-TCTT) (63%) (Supplementary Table S2) (Chen et al., 2017; Li et al., 2018; Yang et al., 2020), while the efficacy of thalidomide in TDT patients did not show any statistically significant relationship with XmnI polymorphism or CD41-42 (-TCTT) mutation (*p* > 0.05) (Supplementary Table S3). In the future, further large sample RCTs are needed to demonstrate the efficacy of thalidomide in TDT patients. After thalidomide treatment, although most AEs were mild and tolerable, considering the serious AEs of thalidomide such as embryotoxicity, careful consideration should be given to the use of thalidomide in young women and children, and to ensure that women have strict contraception during treatment. In addition, clinical symptoms of patients with *ß*-thalassemia were significantly improved, while in most studies the effect of thalidomide on Hb and HbF level appeared to be rather modest. Regarding the mechanisms of thalidomide in *ß*-thalassemia are still unclear, and Chen et al. demonstrated that thalidomide might not only have efficacy in HbF but also affect erythropoiesis at multiple stages (Chen et al., 2021). So it is necessary to extract the effective components of thalidomide and identify the key mechanisms of thalidomide in the effective treatment of thalassemia. Pomalidomide, a third-generation immunomodulatory drug with less AEs, has been proved to be a strong HbF inducer (Khamphikham et al., 2020). If more effective and less embryotoxic thalidomide derivatives are developed, it will be widly used and benefit more thalassemia patients. To sum up, it is the first meta-analysis that confirmed the efficacy and safety of thalidomide preliminarily, which could improve prognosis and quality of life in patients with *ß* -thalassemia by increasing Hb level.

Regarding the possible mechanisms of thalidomide inducing the expression of *γ*-globin gene, a number of studies have shown that it might enhance erythroid transcription factors (e.g., GATA-binding factor 1 (GATA-1)) and inhibit inflammatory factors (e.g., tumor necrosis factor-α (TNF-α)) to increase the production of intracellular reactive oxygen species (ROS) (Amirshahrokhi and Khalili, 2015; Jalali Far et al., 2016). Then, increased production of ROS-mediated p38 MAPK signaling and histone H4 acetylation induces *γ*-globin gene expression (Aerbajinai et al., 2007). Furthermore, it has been reported that thalidomide could effectively induce γ-globin by promoting the proliferation of immature erythroid cells and slowing down erythroid differentiation (Parseval et al., 2008). Recently, *in vitro* studies using the thalidomide derivative pomalidomide suggested that reduced expression of the transcriptional repressor BCL11A could improve the expression of HbF (Dulmovits et al., 2016; Peslak et al., 2020). With the increase of HbF level, the formation of free *a*-globin chain decreases, alleviating the imbalanced relationship between *a*-globin chain and *ß*-globin chain in *ß*-thalassemia patients, so as to improve the clinical symptoms of *ß*-thalassemia patients.

The present study contains a few limitations. First, most studies included in this meta-analysis were single-arm trials with a small sample size, and almost half studies were conducted in China. Second, there was a moderate-to-severe heterogeneity among the included studies. Considering that there was no significant correlation between the genotype and phenotype of *ß*-thalassemia, as well as blood transfusion requirements, other factors, which were not assessed in this study, might be the source of heterogeneity. Third, we did not observed a statistically significant change in SF and spleen length in this meta-analysis. Last but not least, the optimal maintenance dose and median thalidomide treatment time remained elusive. Thus, further long-term, and high-quality prospective trials are required to eliminate the above-mentioned deficiencies and to confirm long-term safety and efficacy of thalidomide more reliably.

## Conclusion

In summary, we, for the first time, conducted this meta-analysis to evaluate the efficacy and safety of thalidomide in the treatment of patients with *ß*-thalassemia. The results showed that thalidomide could be a relatively safe and evidently effective therapy to reduce the blood transfusion requirements and increase Hb level in patients with *ß*-thalassemia, confirming its role as a promising HbF inducer. Extracting the effective components of thalidomide and identify the key mechanisms of thalidomide in the effective treatment of thalassemia will improve its clinical use in *ß*-thalassaemia management.

## Data Availability

The original contributions presented in the study are included in the article/Supplementary Material, further inquiries can be directed to the corresponding authors.
