# Supplementary material for: Investigating the Efficacy and Safety of Thalidomide for Treating Patients With ß-Thalassemia: A Meta-Analysis
Source: Front Pharmacol. 2022 Jan 11;12:814302. doi: 10.3389/fphar.2021.814302 (PMC8786914; doi:10.3389/fphar.2021.814302)
Supplement: Supplementary file 1 [file DataSheet1.docx]

**Figure 1. Flowchart of study selection process. NOS, Newcastle-Ottawa Scale.**

**Figure 2. A forest plot illustrating overall response rate in population-based subgroups.**

**Figure 3. A forest plot illustrating complete response rate in population-based subgroups.**

**Figure 4. A forest plot illustrating Hb level (g/dL) after the treatment of thalidomide in population-based subgroups.**

**Figure 5. A forest plot illustrating HbF level (%) after the treatment of thalidomide.**

**Figure 6. A forest plot illustrating adverse effects of thalidomide.**

**
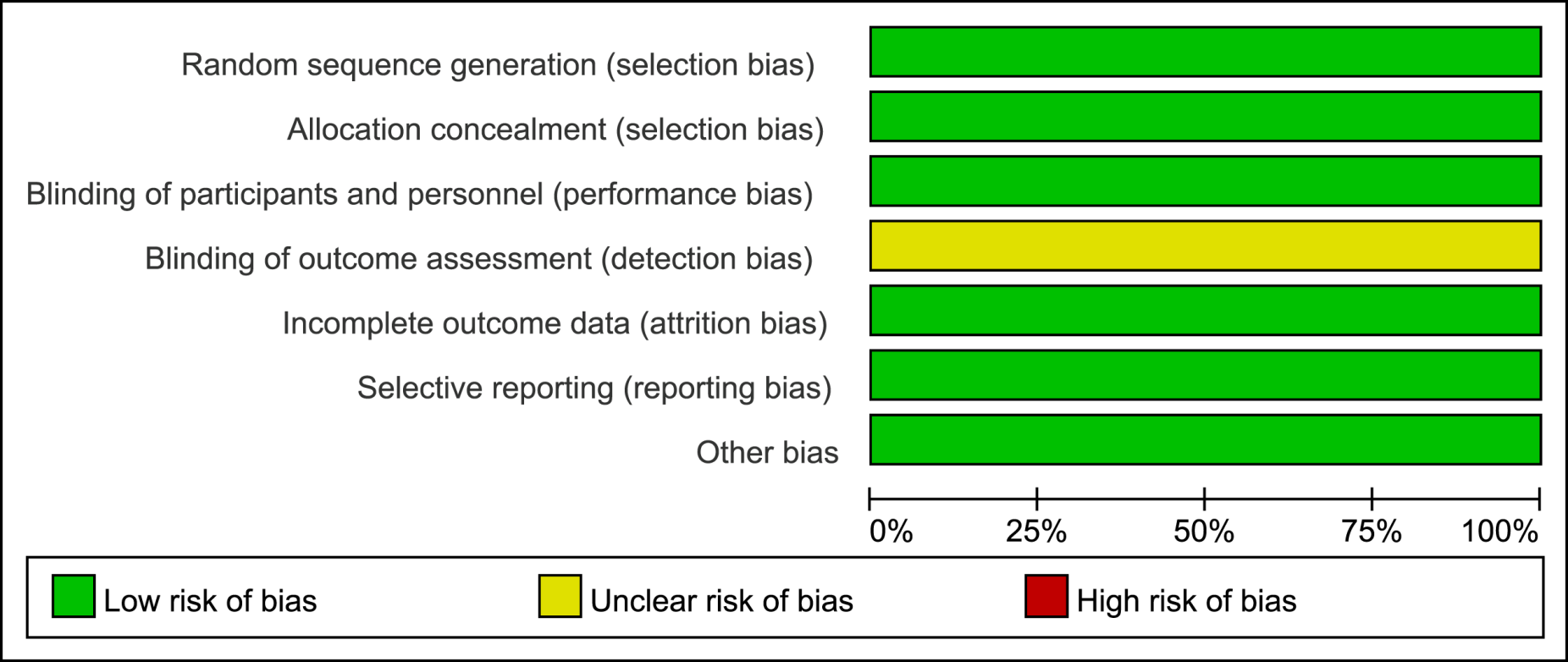
**

**Figure S1. The Cochrane Collaboration risk assessment tool used to assess the risk of bias of the randomized controlled trials.**

**
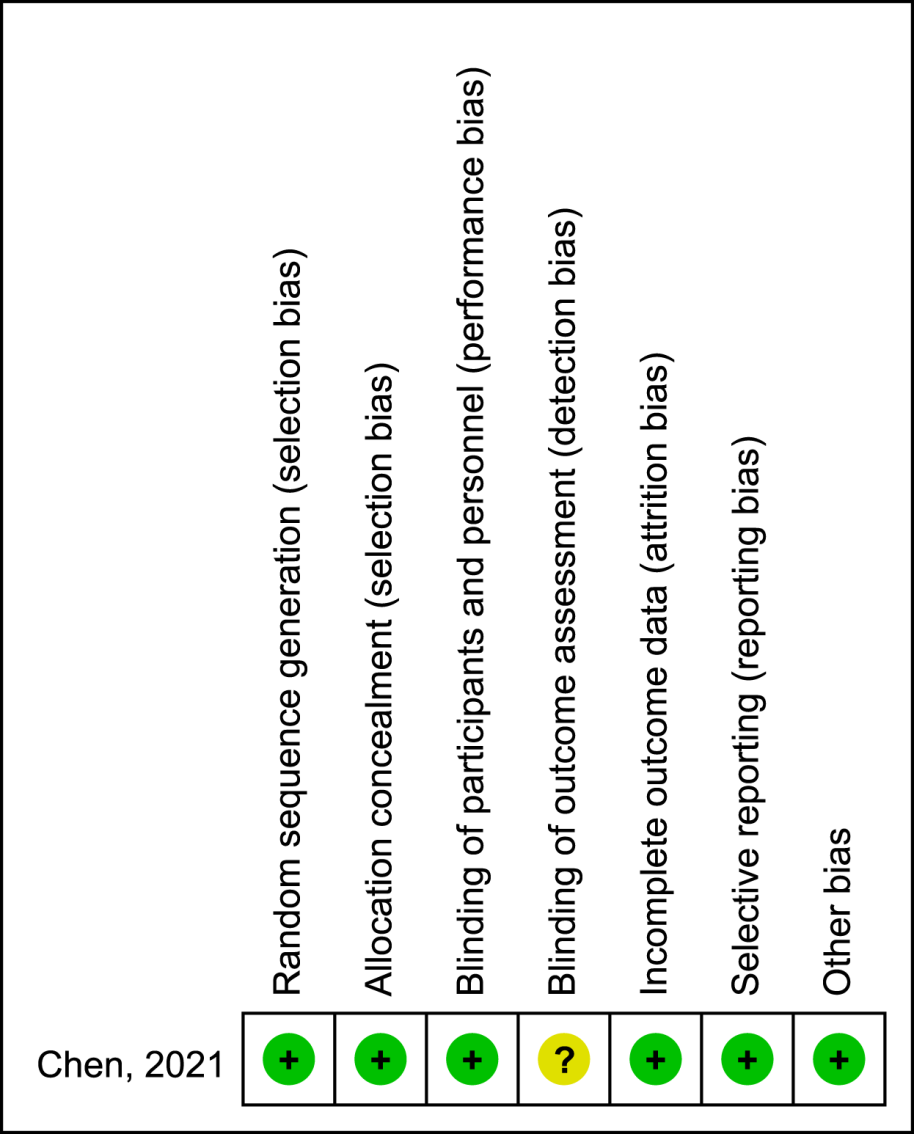
**

**Figure S2. The Cochrane Collaboration risk assessment tool used to assess the risk of bias summary of the randomized controlled trials.**

**
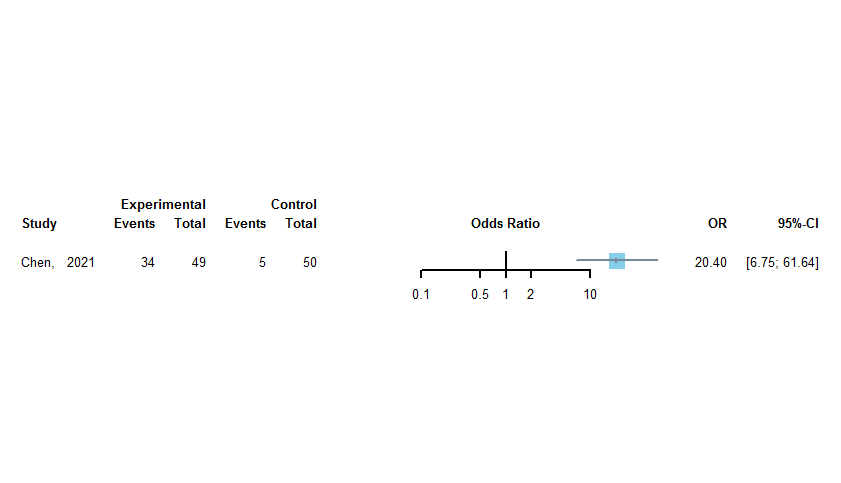
**

**Figure S3. A forest plot illustrating overall response rate comparing thalidomide group with placebo group.**

**
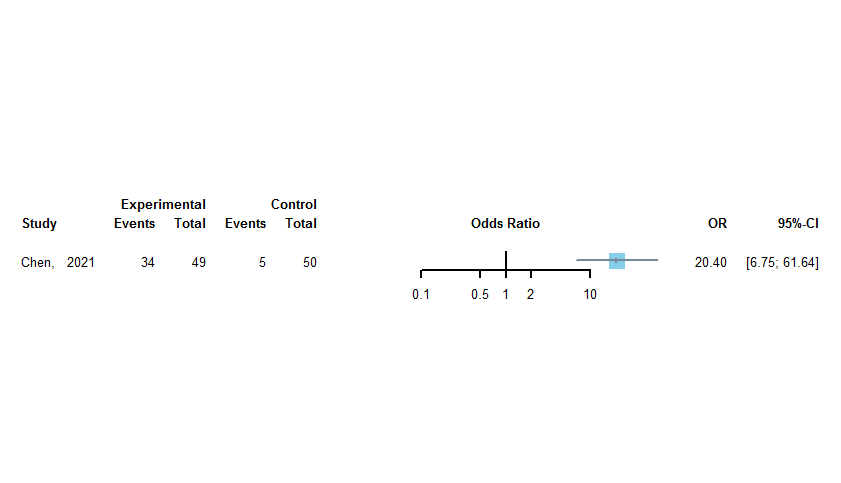
**

**Figure S4. A forest plot illustrating complete response rate comparing thalidomide group with placebo group.**

**
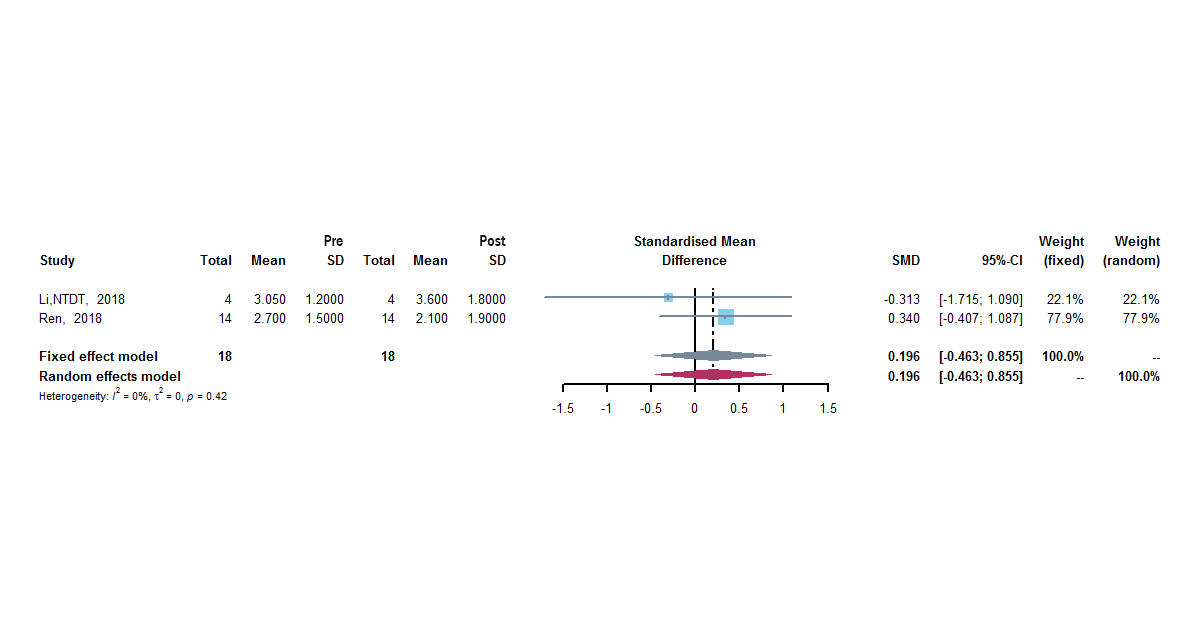
**

**Figure S5. A forest plot illustrating HbA level (g/dL) after the treatment of thalidomide.**

**
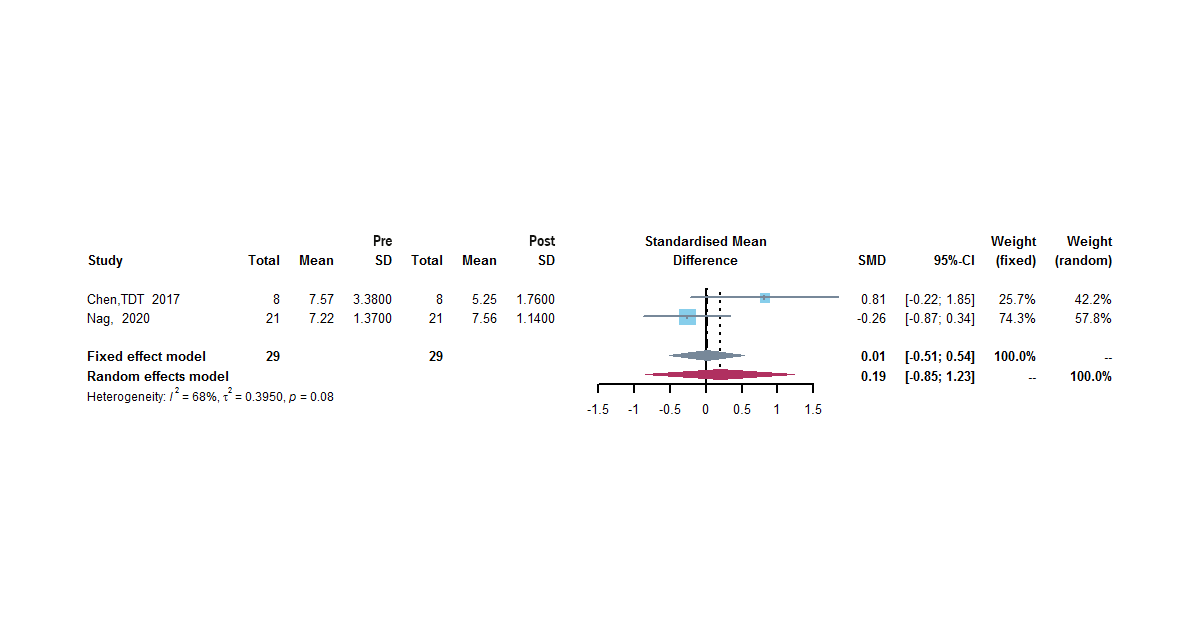
**

**Figure S6. A forest plot illustrating spleen length (cm) after the treatment of thalidomide.**

**
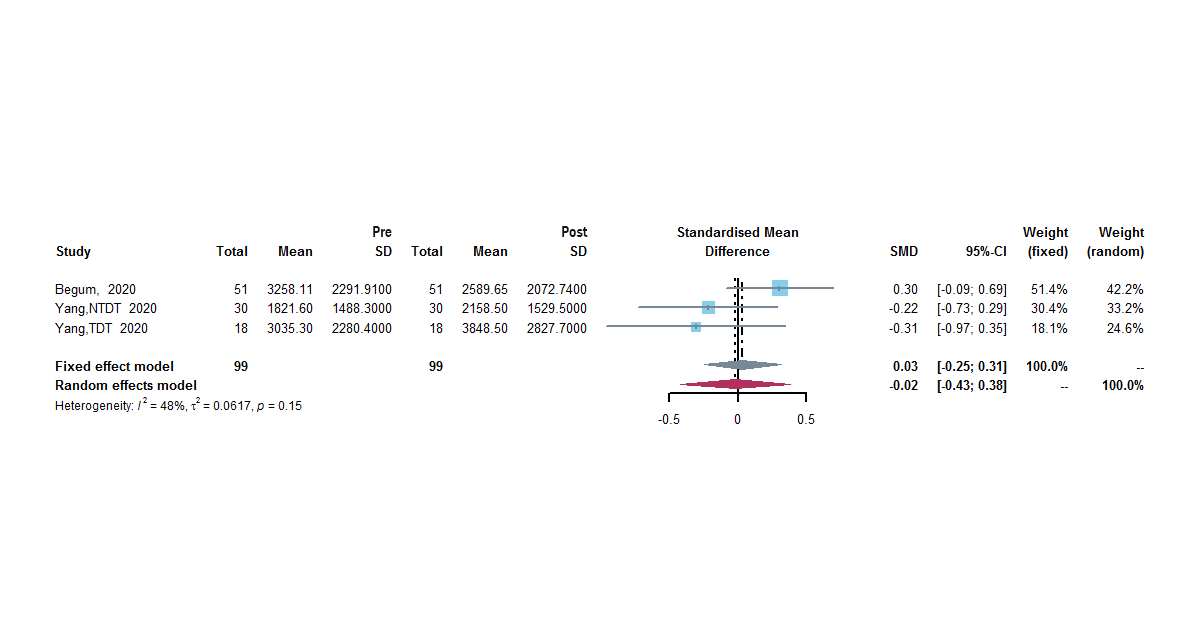
**

**Figure S7. A forest plot illustrating serum ferritin level (ng/ml) after the treatment of thalidomide.**

**
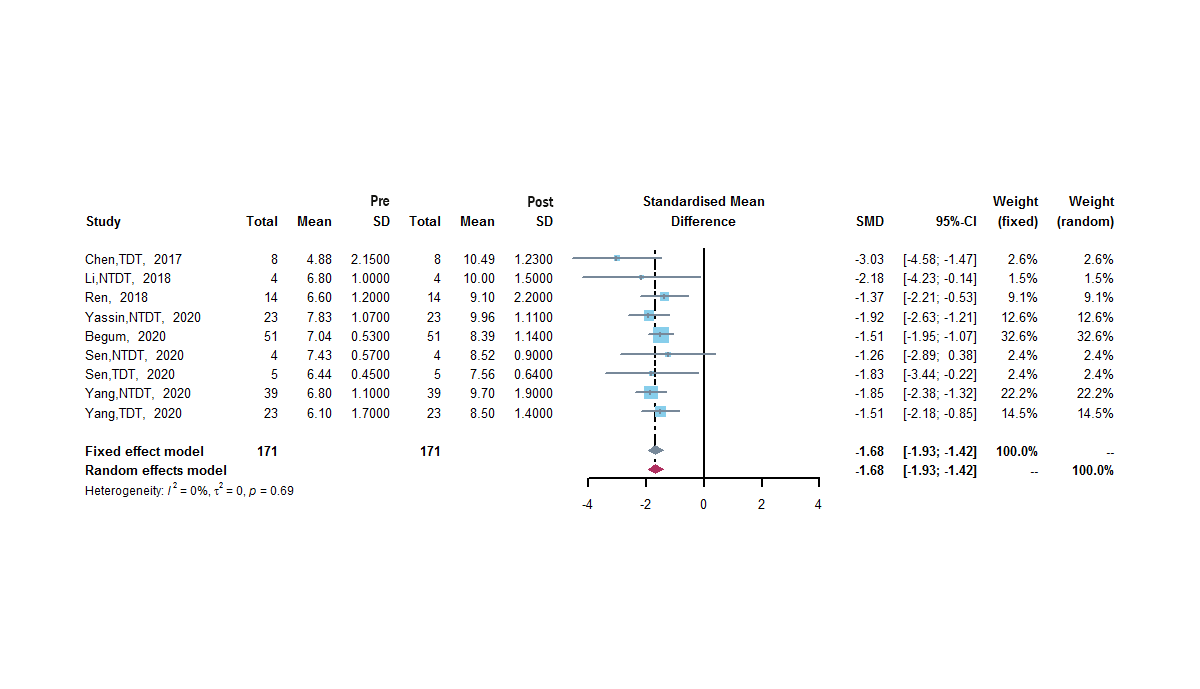
**

**Figure S8. A forest plot illustrating sensitivity analysis of Hb level after exclusion of Nag et al.’s study.**

**Table S1. NOS used to assess the quality of the single-arm studies**

|  | Selection |  |  |  | Comparability | Outcome |  |  | | | | |  |
| --- | --- | --- | --- | --- | --- | --- | --- | --- | --- | --- | --- | --- | --- |
| Study | Representativeness of the exposed cohort | Selection of the non exposed cohort | Ascertainment of exposure | Demonstration that outcome of interest was not present at start of study | Comparability of cohorts on the basis of the design or analysis | Assessment of outcome | Was follow-up long enough for outcomes to occur | | Adequacy of follow up of cohorts | | Total score |  |  |
| Chen, 2017 |  |  |  |  |  |  |  |  | | 5 | | | |
| Li, 2018 |  |  |  |  |  |  |  |  | | 6 | | | |
| Ren, 2018 |  |  |  |  |  |  |  |  | | 7 | | | |
| Jain, 2020 |  |  |  |  |  |  |  |  | | 8 | | | |
| Yassin, 2020 |  |  |  |  |  |  |  |  | | 6 | | | |
| Nag, 2020 |  |  |  |  |  |  |  |  | | 5 | | | |
| Islam, 2020 |  |  |  |  |  |  |  |  | | 5 | | | |
| Begum, 2020 |  |  |  |  |  |  |  |  | | 5 | | | |
| Sen, 2020 |  |  |  |  |  |  |  |  | | 5 | | | |
| Yang, 2020  Li, 2021 |  |  |  |  |  |  |  |  | | 7  6 | | | |

**Table S2. Genotypes and outcomes in 30 β-thalassemia patients**

| **Genotype** | **Outcomes** | | | **Total**  **N^*^ = 30** |
| --- | --- | --- | --- | --- |
|  | **CRR**  **N^*^ = 21** | **ORR**  **N* = 8** | **NR**  **N^*^ = 1** |  |
| β^CD41-42^/β^-28^  β^CD41-42^/β^βE^  β^CD41-42^/ β^CD41-42^  β^CD17^/β^-28^  β^CD41-42^/β^N^  β^CD17^/β^-29^  β^CD41-42^/β^CD17^  β^654^/β^βE^  β^IVSⅡ-654^/β^N^  β^CD43^/β^βE^  β^28^/β^-28^  β^CD17^ /β^βE^  β^CD17^/N | 4  3  3  3  1  2  0  1  1  1  1  1  0 | 3  2  0  0  2  0  1  0  0  0  0  0  0 | 0  0  0  0  0  0  0  0  0  0  0  0  1 | 7  5  3  3  3  2  1  1  1  1  1  1  1 |

*N, normal; CD, codon; N****^*^****, sample size; ORR, overall response rate; CRR, complete response rate; NR, no response.*

**Table S3. Clinical responses to thalidomide in 30 β-thalassemia patients regarding XmnI polymorphism and CD41-42 (-TCTT) mutation**

|  | **XmnI** | | | | **CD41-42 (-TCTT) homozygous/**  **heterozygous** | | **CD41-42 (-TCTT) homozygous** | |
| --- | --- | --- | --- | --- | --- | --- | --- | --- |
|  | **β+/βN & β0/βN β+/β+ β0/β+ β0/β0** | | | | **Yes** | **No** | **Yes** | **No** |
| RR (N^*^) |  | 4 | 3 | 18 4 | 19 | 10 | 3 | 26 |
| NR (N^*^)  *P value* |  | 1 | 0  0.4 | 0 0 | 0  0.367 | 1 | 0  1 | 1 |

*N, normal; N, sample size; RR, response to thalidomide; NR, no response to thalidomide. CD41-42 (-TCTT) homozygous/ heterozygous, patients with CD41-42 (-TCTT) homozygous mutation or CD41-42 (-TCTT) heterozygous mutation; CD41-42 (-TCTT) homozygous, patients with CD41-42 (-TCTT) homozygous mutation.* We categorized all 30 patients in four groups that were β+/βN and β0/βN, β+/β+, β0/β+ and β0/β0 mutations.
